# Molecular epidemiology of β-lactamases in ceftriaxone-resistant Enterobacterales bloodstream infections in the mid-Atlantic United States

**DOI:** 10.1128/aac.01258-24

**Published:** 2025-01-27

**Authors:** Dariusz A. Hareza, Yehudit Bergman, Emily Jacobs, Jennifer Lu, Nancy D. Hanson, Rick Conzemius, Sara E. Cosgrove, Anthony D. Harris, Patricia J. Simner, Pranita D. Tamma

**Affiliations:** 1 Department of Medicine, Johns Hopkins University School of Medicine 1500 , Baltimore, Maryland, USA; 2 Department of Pathology, Johns Hopkins University School of Medicine 1500 , Baltimore, Maryland, USA; 3 Department of Pediatrics, Johns Hopkins University School of Medicine 1500 , Baltimore, Maryland, USA; 4 Department of Microbiology and Immunology, Creighton School of Medicine 12282 , Omaha, Nebraska, USA; 5 Ares Genetics, Vienna, Austria; 6 Department of Epidemiology and Public Health, University of Maryland School of Medicine 12264 , Baltimore, Maryland, USA; University of California, San Francisco, San Francisco, California, USA

**Keywords:** ESBLs, CTX-M, SHV, *ampC*, CMY, DHA, EC

## Abstract

Ceftriaxone-resistant Enterobacterales remain a public health threat; contemporary data investigating their molecular epidemiology are limited. Five hundred consecutive ceftriaxone-resistant (MIC ≥ 4 µg/mL) Enterobacterales bloodstream isolates were collected between 2018 and 2022 from three Maryland hospitals. Broth microdilution confirmed antibiotic susceptibilities. Whole-genome sequencing identified extended-spectrum β-lactamase (ESBL) and *ampC* genes both in bacterial chromosomes (c-*ampC*) and on plasmids (p-*ampC*). Mutations in promoter or attenuator regions of the *Escherichia coli* c-*ampC* gene (i.e., *bla*_EC_ gene) with the potential to result in *ampC* derepression were investigated. The presence of ESBL or *ampC* genes was confirmed in 497 (99.4%) isolates. Two hundred seventy-nine (55.8%) isolates had both ESBL and *ampC* genes. ESBL families were identified among 398 (80%) patients: *bla*_CTX-M_ (*n* = 370), *bla*_SHV_ (*n* = 17), *bla*_OXY_ (*n* = 14), and *bla*_VEB_ (*n* = 5). Ceftriaxone-resistant Enterobacterales species carrying ESBL genes included the following: *E. coli* (67%), *Klebsiella pneumoniae* (24%), *Klebsiella oxytoca* (4%), *Proteus mirabilis* (2%), *Enterobacter cloacae* complex (2%), *Klebsiella aerogenes* (1%), *Providencia stuartii* (<1%), and *Serratia marcescens* (<1%). c-*ampC* genes were identified in 374 (75%) of the 500 isolates. Only 7% of *E. coli* isolates with mutations in the promoter or attenuator region of the c-*ampC* gene exhibited resistance to cefoxitin, a proxy for increased AmpC production. Two p-*ampC* genes were confirmed in 25 (5%) of the 500 isolates: *bla*_CMY-59_ (72%) and *bla*_DHA-1_ (28%; confined to *E. coli* [92%] and *K. pneumoniae* [8%]). Until comprehensive β-lactamase molecular testing is available, the species-specific prevalence of ESBL and *ampC* genes in ceftriaxone-resistant Enterobacterales should be considered to promote effective albeit judicious antibiotic prescribing. Mutations in promoter or attenuator regions of the *E. coli* c-*ampC* gene do not appear to contribute significantly to increased AmpC production in this species.

## INTRODUCTION

The incidence of ceftriaxone-resistant Enterobacterales infections continues to increase in the United States, while rates of other antimicrobial-resistant organisms (e.g., carbapenem-resistant Enterobacterales, carbapenem-resistant *Acinetobacter baumannii*, and multidrug-resistant *Pseudomonas aeruginosa*) have remained relatively stable (1). However, surprisingly little is known about the molecular epidemiology of β-lactamase genes in ceftriaxone-resistant Enterobacterales (i.e., extended-spectrum β-lactamase [ESBL] or *ampC* genes), in contrast to what is known about the contemporary epidemiology of carbapenemase genes in carbapenem-resistant Enterobacterales, despite the significantly higher frequency of the former compared to the latter.

In the absence of ESBL testing, many hospitals use *Escherichia coli*, *Klebsiella oxytoca*, *Klebsiella pneumoniae*, and *Proteus mirabilis* non-susceptible results to third-generation cephalosporins as a proxy for ESBL production (2). This approach has limitations for two primary reasons. First, ESBL production is not confined to these four bacterial species and, rather, can be produced by any Enterobacterales (3). Second, although ceftriaxone non-susceptible results for these four species are sensitive for detecting ESBL production, this criteria has limitations with specificity (4). To circumvent issues with phenotypic ESBL detection, several United States Food and Drug Administration-cleared diagnostic platforms have become available in recent years for ESBL gene identification directly from clinical specimens. These molecular assays, however, are limited to *bla*_CTX-M_ genes (3, 5, 6), which are the most common ESBL genes but not the only ESBL genes.

The situation is arguably even more complicated for *ampC* genes as accurate and practical phenotypic and genotypic approaches for their detection in clinical microbiology laboratories are lacking. Increased AmpC production by Enterobacterales generally occurs by one of three mechanisms: (i) inducible chromosomal *ampC* gene expression (e.g., increased expression of *ampC* in *Enterobacter cloacae* complex in response to exposure to certain β-lactam agents), (ii) stable chromosomal *ampC* gene de-repression (e.g., mutations in promoter or attenuator regions of the chromosomal *ampC* gene of *E. coli* [i.e., *bla*_EC_ genes] that lead to increased AmpC production and to ceftriaxone resistance), or (iii) constitutively expressed *ampC* genes that are frequently present on plasmids (p-*ampC*) and sometimes integrated into the chromosome (c-*ampC*) of bacteria (e.g., the p-*ampC* gene *bla*_CMY_ in *E. coli* or the c-*ampC* gene *bla*_CMY_ in *Citrobacter freundii*).

A comprehensive understanding of β-lactamase molecular epidemiology in ceftriaxone-resistant Enterobacterales is important to inform treatment decisions since the preferred treatment of ESBL-E infections differs from AmpC-producing Enterobacterales (AmpC-E) infections. More specifically, carbapenems are considered the preferred therapy for ESBL-E bloodstream infections, whereas cefepime is generally considered first-line therapy for AmpC-E bloodstream infections (2). Understanding when ceftriaxone-resistant Enterobacterales species are unlikely to contain an ESBL gene can be a useful carbapenem-sparing approach. We sought to understand comprehensive ESBL and *ampC* gene prevalence, species-specific β-lactamase epidemiology, and associations between β-lactamase genes and antimicrobial susceptibility testing (AST) results to inform interim antibiotic decision-making while awaiting future, more comprehensive β-lactamase molecular diagnostic panels.

## MATERIALS AND METHODS

### Microbiological methods

Five hundred consecutive ceftriaxone-resistant (MIC ≥ 4 µg/mL) Enterobacterales bloodstream isolates were collected from 2018 to 2022 from unique patients at three Maryland hospitals. Bacterial genus and species identification occurred using matrix-assisted laser-desorption ionization time-of-flight mass spectrometry (Bruker Daltonics Inc., Billerica, Massachusetts). Initial AST was performed by the BD Phoenix Automated System (BD Diagnostics, Sparks, Maryland) gram-negative NMIC-303 or NMIC-306 panels. AST results were confirmed using lyophilized Sensititer broth microdilution (BMD) GN2F panels (Thermo Fisher Scientific, Waltham, Massachusetts); disk diffusion testing was used to determine ertapenem susceptibility (BD Diagnostics). Clinical and Laboratory Standards Institute interpretive criteria were applied to determine susceptibility (7). Susceptible dose-dependent (SDD) interpretations for Enterobacterales isolates against cefepime and piperacillin-tazobactam were categorized as susceptible, given anticipated clinical treatment success with increased antibiotic dosages and infusion durations (7). Isolates had to be susceptible to ertapenem, imipenem, and meropenem to be included in the cohort.

### Whole-genome sequencing

Genomic DNA extraction was performed using the PowerSoil Kit (QIAGEN, Valencia, California). Whole-genome sequencing (WGS) was performed using Illumina 300 bp paired-end sequencing (2 × 150 bp; P2) on the Illumina NextSeq 1000 (Illumina, San Diego, California). AREScloud release 2022–10 (Ares Genetics, Vienna, Austria) was used for genome assembly, organism identification, sequence type (ST), plasmid type, and antimicrobial resistance gene detection (8). DIAMOND v1.0.11 was performed on six-frame translated genome assemblies against ARESdb with 60% minimum query coverage and 90% identity (9, 10) for additional AMR gene identification. The NCBI Reference Gene Catalog was used to annotate β-lactamase genes (11). ESBL and *ampC* genes were identified according to published resources (12–14). In addition to the presence and location of *ampC* genes (i.e., bacterial chromosome or plasmid), mutations in the promoter or attenuator regions of the c-*ampC* gene of *E. coli* were investigated using *E. coli* K-12 as the reference (15–20). The location of mutations in the promoter and attenuator region was identified between the −141 and +81 region of the *ampC* gene, based on previously published numbering of bases within the gene (17, 21). The study was approved by The Johns Hopkins University School of Medicine Institutional Review Board. A waiver of informed consent was granted.

## RESULTS

The presence of ESBL or *ampC* genes was confirmed in 497 (99.4%) isolates; 279 (55.8%) isolates had both an ESBL gene and an *ampC* gene. Of the 500 consecutive ceftriaxone-resistant Enterobacterales blood culture isolates obtained from unique patients, the following species were identified: *C. freundii* (8, 1.6%), *E. cloacae* (51, 10.2%), *E. coli* (287, 57.4%), *Klebsiella aerogenes* (18, 3.6%), *K. oxytoca* (14, 2.8%), *K. pneumoniae* (100, 20%), *Morganella morganii* (2, 0.4%), *P. mirabilis* (9, 1.8%), *Proteus penneri* (1, 0.2%), *Providencia stuartii* (1, 0.2%), and *Serratia marcescens* (9, 1.8%). Details about the bacterial species, ST, plasmids identified, and AMR markers for the 500 ceftriaxone-resistant Enterobacterales isolates are available in the supplemental material.

### ESBL gene overview

Of the 500 ceftriaxone-resistant Enterobacterales blood culture isolates, 398 (79.6%) were confirmed to have ESBL genes (Table 1). Four ESBL gene families were identified among 398 patients: *bla*_CTX-M_ (*n* = 370), *bla*_SHV_ (*n* = 17), *bla*_OXY_ (*n* = 14), and *bla*_VEB_ (*n* = 5). Eight Enterobacterales isolates contained two different ESBL genes. Common (i.e., >10% prevalence per ESBL family) *bla*_CTX-M_ genes included *bla*_CTX-M-15_ (282, 76.2%) and *bla*_CTX-M-27_ (54, 14.6%). *bla*_CTX-M_ was the only ESBL gene family found among *E. coli*.

**TABLE 1:** ESBL and p-*ampC* genes identified in 500 ceftriaxone-resistant Enterobacterales bloodstream infections at three Maryland hospitals from 2018 to 2022

ESBL or p-*ampC* Gene^*a*^^,^ ^*b*^	Organism											
	Total (*n*)	*C. freundii* (*n* = 8)	*E. cloacae* complex^*c*^ (*n* = 51)	*E. coli* (*n* = 287)	*K. aerogenes* (*n* = 18)	*K. oxytoca* (*n* = 14)	*K. pneumoniae* (*n* = 100)	*M. morganii* (*n* = 2)	*P. mirabilis* (*n* = 9)	*P. penneri* (*n* = 1)	*P. stuartii* (*n* = 1)	*S. marcescens* (*n* = 9)
*bla*_CTX-M-type_ only	356	0 (0%)	5 (10%)	259 (90%)	3 (17%)	0 (0%)	85 (85%)	0 (0%)	3 (33%)	0 (0%)	1 (100%)	0 (0%)
*bla*_CTX-M-1_	1	0	0	1	0	0	0	0	0	0	0	0
*bla*_CTX-M-2_	2	0	0	0	0	0	0	0	1	0	1	0
*bla*_CTX-M-3_	5	0	0	2	0	0	3	0	0	0	0	0
*bla*_CTX-M-14_	17	0	0	16	0	0	1	0	0	0	0	0
*bla*_CTX-M-15_	269	0	5	180	3	0	80	0	1	0	0	0
*bla*_CTX-M-27_	53	0	0	52	0	0	1	0	0	0	0	0
*bla*_CTX-M-55_	8	0	0	8	0	0	0	0	0	0	0	0
*bla*_CTX-M-65_	1	0	0	0	0	0	0	0	1	0	0	0
*bla*_SHV-type_ only	14	0 (0%)	3 (6%)	0 (0%)	1 (6%)	0 (0%)	9 (9%)	0 (0%)	0 (0%)	0 (0%)	0 (0%)	1 (11%)
*bla*_SHV-2_	3	0	0	0	0	0	3	0	0	0	0	0
*bla*_SHV-7_	5	0	1	0	1	0	2	0	0	0	0	1
*bla*_SHV-12_	2	0	2	0	0	0	0	0	0	0	0	0
*bla*_SHV-30_	1	0	0	0	0	0	1	0	0	0	0	0
*bla*_SHV-39_	1	0	0	0	0	0	1	0	0	0	0	0
*bla*_SHV-187_	2	0	0	0	0	0	2	0	0	0	0	0
*bla*_OXY-type_ only	8	0 (0%)	0 (0%)	0 (0%)	0 (0%)	8 (57%)	0 (0%)	0 (0%)	0 (0%)	0 (0%)	0 (0%)	0 (0%)
*bla*_OXY-2-1_	1	0	0	0	0	1	0	0	0	0	0	0
*bla*_OXY-2-2_	1	0	0	0	0	1	0	0	0	0	0	0
*bla*_OXY-2-4_	4	0	0	0	0	4	0	0	0	0	0	0
*bla*_OXY-2-7_	1	0	0	0	0	1	0	0	0	0	0	0
*bla*_OXY-2-11_	1	0	0	0	0	1	0	0	0	0	0	0
*bla*_VEB-type_ only	5	0 (0%)	0 (0%)	0 (0%)	0 (0%)	0 (0%)	0 (0%)	0 (0%)	5 (56%)	0 (0%)	0 (0%)	0 (0%)
*bla*_VEB-6_	5	0	0	0	0	0	0	0	5	0	0	0
Total ESBL isolates	398	0 (0%)	8 (16%)	265^*a*^ (92%)	4 (22%)	14^*a*^ (100%)	97^*a*^ (97%)	0 (0%)	8 (89%)	0 (0%)	1 (100%)	1 (11%)
*bla*_CMY-59_ only	15	0	0	15	0	0	0	0	0	0	0	0
*bla*_DHA-1_ only	3	0	0	2	0	0	1	0	0	0	0	0
Total p-*ampC*	25	0 (0%)	0 (0%)	23 (8%)	0 (0%)	0 (0%)	2 (2%)	0 (0%)	0 (0%)	0 (0%)	0 (0%)	0 (0%)

^*a*^ Isolates with multiple ESBL and/or p-*ampC* genes: *bla*_CTX-M-15_ + *bla*_OXY-1-1_, *K. oxytoca* (4); *bla*_CTX-M-15_ + *bla*_OXY-1-7_, *K. oxytoca* (1); *bla*_CTX-M-15_ + *bla*_SHV-187_, *K. pneumoniae* (2); *bla*_CTX-M-15_ + *bla*_CMY-59_, *E. coli* (3); *bla*_CTX-M-15_ + *bla*_DHA-1_, *E. coli* (2), *K. pneumoniae* (1); *bla*_CTX-M-27_ + *bla*_DHA-1_, *E. coli* (1); *bla*_OXY-1-1_ + *bla*_SHV-7_, *K. oxytoca* (1).

^*b*^ Includes eight isolates with more than one ESBL gene and seven isolates with an ESBL and p-*ampC* gene.

^*c*^ Of the eight *E. cloacae* complex isolates with ESBL genes, ESBL genes were present in *Enterobacter hormaechei* (7) and *Enterobacter roggenkampii* (1).

Common *bla*_SHV_ ESBL genes included *bla*_SHV-7_ (6, 35.3%), *bla*_SHV-187_ (4, 23.5%), *bla*_SHV-2_ (3, 17.6%), and *bla*_SHV-12_ (2, 11.8%). Common *bla*_OXY_ genes among *K. oxytoca* included *bla*_OXY-1-1_ (5, 35.7%) and *bla*_OXY-2-4_ (4, 28.6%). All five *bla*_VEB_ genes were *bla*_VEB-6_ and found only among *P. mirabilis*. Enterobacterales species carrying ESBL genes included the following (out of a denominator of 398 isolates): *E. coli* (265, 66.5%), *K. pneumoniae* (97, 24.4%), *K. oxytoca* (14, 3.5%), *P. mirabilis* (8, 2%), *E. cloacae* complex (8, 2%), *K. aerogenes* (4, 1%), *P. stuartii* (1, < 1%), and *S. marcescens* (1, < 1%).

### c-*ampC* gene overview

Out of the 500 ceftriaxone-resistant Enterobacterales isolates, 377 (75.4%) contained a c-*ampC* gene. All isolates in the following bacterial species harbored c-*ampC* genes: *C. freundii* (*n* = 8), *E. cloacae* complex (*n* = 51), *E. coli* (*n* = 287), *K. aerogenes* (*n* = 18), *M. morganii* (*n* = 2), *P. stuartii* (*n* = 1), and *S. marcescens* (*n* = 9). One of the nine *P. mirabilis* isolates harbored a c-*ampC* gene. For these species, with the exception of *E. coli*, constitutive basal levels of *ampC* expression lead to hydrolysis of some agents (e.g., cefazolin) but generally not others (e.g., ceftriaxone), except when there is increased expression of *ampC* (i.e., *ampC* hyperexpression) in response to exposure to certain β-lactam agents, leading to ceftriaxone hydrolysis (22). For *E. coli*, however, c-*ampC* gene expression generally only results in ceftriaxone resistance (assuming no ESBL or p-*ampC* genes are present) when there are mutations in the promoter or attenuator regions of the c-*ampC* gene (15–19).

**Fig 1:**
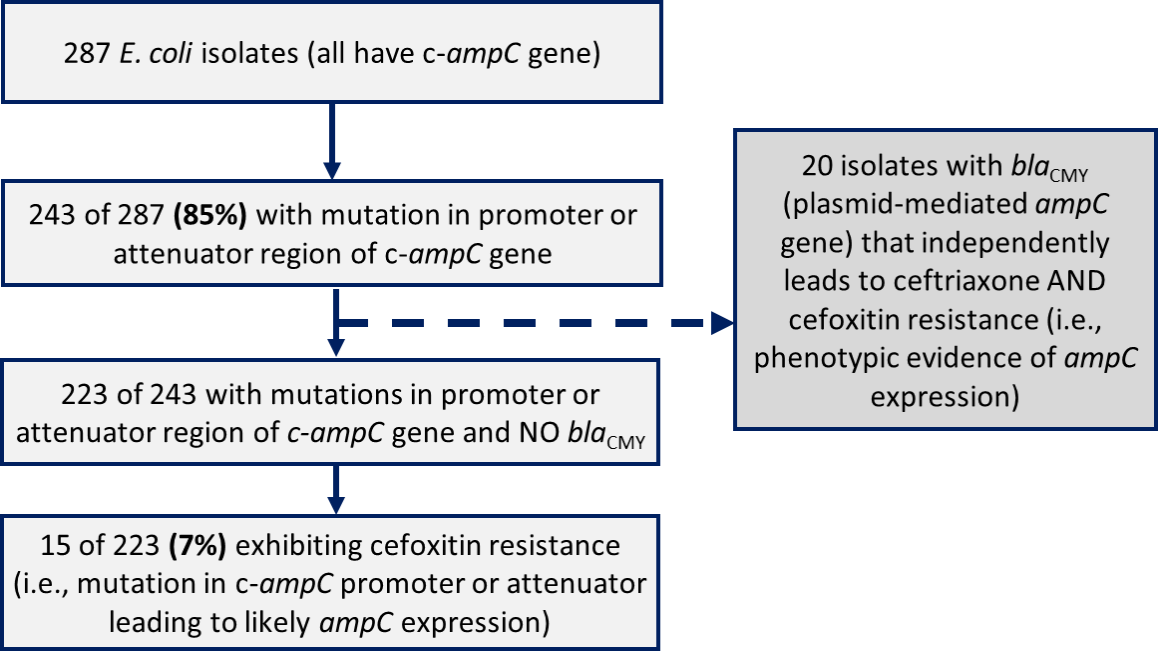
A deeper investigation into c-*ampC* genes in *E. coli.*

Of the 287 ceftriaxone-resistant *E. coli* isolates in our cohort, 243 (84.7%) had mutations within the promoter and/or attenuator region relative to the reference strain *E. coli* K-12 (see the supplemental material). For the 243 *E. coli* isolates with mutations within the promoter or attenuator regions, 20 (8.2%) also contained a *bla*_CMY_ gene on the chromosome or plasmid, making it challenging to determine the contribution of the mutation within the *ampC* gene to increased *ampC* expression. Of the 223 isolates with mutations in the promoter and/or attenuator region and no *bla*_CMY_ genes, only 15 (6.7%) exhibited resistance to cefoxitin, a proxy for excessive AmpC production (Fig. 1).

### *p-ampC* gene overview

Out of the 500 ceftriaxone-resistant Enterobacterales isolates, 25 (5%) contained a p-*ampC* gene. p-*ampC* genes include: *bla*_CMY-59_ (18, 72%) and *bla*_DHA-1_ (7, 28%; Table 1). Enterobacterales species harboring p-*ampC* genes were limited to *E. coli* (23, 92%) and *K. pneumoniae* (2, 8%).

### Enterobacterales harboring β-lactamase genes

The proportion of Enterobacterales bloodstream isolates containing an ESBL gene out of all Enterobacterales bloodstream isolates (i.e., both ceftriaxone-susceptible and non-susceptible) from 2018 to 2022 was then investigated. Out of a total of 2,894 Enterobacterales bloodstream isolates from unique consecutive patients identified during the study period, 13.8% contained ESBL genes. The species-specific ESBL prevalence was as follows: *E. coli* (265/1,056, 25.1%), *K. pneumoniae* (97/804, 12.1%), *K. oxytoca* (14/140, 10%), *P. mirabilis* (8/128, 6.3%), *P. stuartii* (1/18, 5.6%), *K. aerogenes* (4/152, 2.6%), *E. cloacae* complex (8/332, 2.4%), and *S. marcescens* (1/172, 0.6%). No ESBL genes were identified in *C. freundii* (*n* = 80), *M. morganii* (*n* = 7), or *P. penneri* (*n* = 5).

Out of a total of 2,894 Enterobacterales bloodstream isolates from unique consecutive patients identified during the study period (both ceftriaxone-susceptible and non-susceptible), <1% contained p-*ampC* genes. The species-specific p-*ampC* gene prevalence was as follows: *E. coli* (23, 2.2%) and *K. pneumoniae* (2, <1%). No p-*ampC* genes were identified in *C. freundii* (*n* = 80), *E. cloacae* complex (*n* = 332), *K. aerogenes* (*n* = 152), *K. oxytoca* (*n* = 140), *M. morganii* (*n* = 7), *P. stuartii* (*n* = 18), *P. penneri* (*n* = 5), or *S. marcescens* (*n* = 172).

### Antimicrobial susceptibility testing

Percent susceptibilities to select antimicrobials based on resistance genes present are listed in Table 2. In isolates harboring ESBL genes only, AST results (percent susceptibilities) by BMD for antibiotics commonly prescribed for ESBL-producing infections were as follows: piperacillin-tazobactam (87%), nitrofurantoin (75%), cefepime (50%), doxycycline (33%), trimethoprim-sulfamethoxazole (27%), aztreonam (6%), and ciprofloxacin (4%). There were several notable observations of antibiotic susceptibility percentages by ESBL family. For example, susceptibility (or SDD) to piperacillin-tazobactam occurred in 89% of isolates producing CTX-M, 79% producing SHV, 38% producing OXY, and 100% producing VEB enzymes, when limiting the analysis to isolates with a single ESBL family. In contrast, cefepime susceptibility (or SDD) was observed in 48% of isolates producing CTX-M, 93% producing SHV, 100% producing OXY, and 0% producing VEB enzymes.

**TABLE 2:** Percent susceptibility of 500 ceftriaxone-resistant Enterobacterales isolates from three Maryland hospitals from 2018 to 2022 with β-lactamase gene characterization to select antibiotics based on the results of broth microdilution testing^*a*^

Gene (*n*)^*b*^	Piperacillin-tazobactam	Cefepime	Trimethoprim-sulfamethoxazole	Ciprofloxacin	Aztreonam	Nitrofurantoin	Doxycycline
ESBL genes (391)^*c*^	87%	50%	27%	4%	6%	75%	33%
*bla*_CTX-M_ only (356)	89%	48%	24%	3%	6%	76%	33%
*bla*_SHV_ only (14)	79%	93%	86%	7%	0%	43%	60%
*bla*_OXY_ only (8)	38%	100%	100%	38%	0%	100%	100%
*bla*_VEB_ only (5)	100%	0%	0%	0%	0%	0%	0%
p-*ampC* genes (18)	72%	94%	44%	44%	33%	94%	20%
*bla*_CMY-59_ only (15)	80%	93%	67%	47%	40%	100%	25%
*bla*_DHA-1_ only (3)	33%	100%	0%	33%	0%	67%	0%

^*a*^ Clinical and Laboratory Standards Institute (CLSI) breakpoints used to determine susceptibility; intermediate MICs categorized as not susceptible; susceptible dose dependent (SDD) MICs categorized as susceptible.

^*b*^ Isolates containing both ESBL and p-*ampC* genes are not included.

^*c*^ Includes isolates with multiple ESBL genes; therefore, the 391 is higher than the sum of the individual ESBL families in the table.

In isolates harboring only p-*ampC* genes, AST results were as follows: cefepime (94%), nitrofurantoin (94%), piperacillin-tazobactam (72%), ciprofloxacin (44%), trimethoprim-sulfamethoxazole (44%), aztreonam (33%), and doxycycline (20%). The seven isolates that had both an ESBL gene and a p-*ampC* gene had lower susceptibilities to almost all antibiotics compared to isolates with only ESBL or p-*ampC* genes with susceptibilities as follows: nitrofurantoin (71%), piperacillin-tazobactam (29%), doxycycline (20%), cefepime (14%), trimethoprim-sulfamethoxazole (14%), ciprofloxacin (0%), and aztreonam (0%).

## DISCUSSION

We investigated a cohort of 500 consecutive ceftriaxone-resistant Enterobacterales bloodstream isolates from unique patients across three hospitals to advance our understanding of the molecular epidemiology of β-lactamase genes in the mid-Atlantic United States. Overall, ESBL genes were identified in approximately 14% of all Enterobacterales isolates (regardless of ceftriaxone susceptibility) during the study period. When limited to isolates that were ceftriaxone-resistant, 80% contained an ESBL gene. The bacterial species most commonly containing ESBL genes were *E. coli*, *K. pneumoniae*, and *K. oxytoca*. Across all Enterobacterales isolates, less than 1% contained a p*-ampC* gene. Restricting the analysis to ceftriaxone-resistant Enterobacterales, 5% contained a p-*ampC* gene, and these were limited to *E. coli* (92%) and *K. pneumoniae* (8%) isolates. As anticipated, a number of Enterobacterales species (e.g., *E. cloacae* complex, *K. aerogenes*, and *C. freundii*) contained c-*ampC* genes. Mutations in the promoter and/or attenuator region of the chromosomal *ampC* gene of *E. coli* (i.e., *bla*_EC_) were common. However, these mutations did not appear to contribute significantly to ceftriaxone resistance among *E. coli* isolates.

Our results are representative of a single region in the United States (i.e., the mid-Atlantic) and need to be evaluated in the context of other United States estimates. A study was undertaken investigating the prevalence of β-lactamase genes in *E. coli* and *K. pneumoniae* isolates exhibiting non-susceptibility to third- or fourth-generation cephalosporins or aztreonam from 56 United States hospitals from 2016 to 2020 (4). ESBL prevalence was 88%, similar to our findings. Since our cohort included a diverse group of Enterobacterales, we were additionally able to identify *bla*_CTX-M_ genes in *E. cloacae* complex, *K. aerogenes*, *K. oxytoca*, and *P. mirabilis*.

The distribution of specific *bla*_CTX-M_ variants in our cohort is also similar to national estimates (4). ESBL-producing *E. coli* is dominated by a highly successful and virulent international clone belonging to ST131 (23). As the ST131 clone contains *bla*_CTX-M_ gene variants, considerably less attention is given to understanding contemporary estimates of other ESBL genes such as *bla*_SHV_ variants and their associated clinical impact. ESBL SHV variants were first identified in 1985 and arose through a single amino acid substitution in the SHV-1 enzyme, leading to an expansion of the substrate profile to include expanded-spectrum cephalosporins (e.g., ceftriaxone) (24). In our cohort, *bla*_SHV_ ESBL variants were identified in *K. pneumoniae*, *E. cloacae* complex, *K. aerogenes*, and *S. marcescens*.

While WGS of all clinical isolates in real time is currently impractical with existing clinical microbiology infrastructure, we believe our findings underscore the importance of developing accurate molecular methods for identification of expanded-spectrum β-lactamase genes rather than relying solely on ceftriaxone non-susceptibility for several reasons. First, relying on ceftriaxone non-susceptibility may contribute to carbapenem overuse. A portion of Enterobacterales with ceftriaxone MICs ≥ 2 µg/mL (even if limited to *E. coli*, *K. pneumoniae*, *K. oxytoca*, or *P. mirabilis*) will not harbor ESBL genes. In our cohort, 6% of ceftriaxone-resistant isolates (limited to *E. coli*, *K. pneumoniae*, *K. oxytoca*, or *P. mirabilis*) did not harbor ESBL genes; this percentage likely would have been higher if less stringent criteria, such as ceftriaxone non-susceptibility as a proxy for ESBL production, had been applied (25). In a study that included 5,723 *E. coli*, *Klebsiella* spp., and *P. mirabilis* isolates with ceftriaxone MICs of ≥2 µg/mL, 13% did not contain ESBL genes (26). We previously performed a hypothetical investigation to explore the impact of reporting ESBL status vs ceftriaxone susceptibility results on carbapenem use (27). If reporting ESBL production from all bacterial specimens (e.g., blood and urine) in our hospital, rather than having clinicians make “*ad hoc*” decisions based on ceftriaxone non-susceptible results as a proxy for ESBL production, carbapenem use could be considerably reduced (27).

A second point in support of ESBL testing is that most clinical microbiology laboratories rely on non-reference methods such as automated susceptibility platforms (e.g., Vitek 2, Phoenix, and MicroScan) to determine antibiotic MICs. Two large studies have previously highlighted the notable overcalling and undercalling of β-lactam MICs with the use of non-reference MIC approaches (28, 29). A total reliance on ceftriaxone susceptibility when derived from non-reference methods to infer ESBL status, as is currently the case, likely results in higher percentages of ESBL misclassification.

Third, although our results support current guidance from the Infectious Diseases Society of America highlighting the concern for ESBL production in ceftriaxone non-susceptible *E. coli*, *K. pneumoniae*, *K. oxytoca*, and *P. mirabilis* (2), 4% of ESBLs identified in our cohort were produced by other Enterobacterales species. This may have clinical implications as these patients would be unlikely to receive carbapenem therapy, which has been shown to be the treatment of choice for ESBL-E infections given the association of carbapenems with increased patient survival (28) (potentially regardless of the specific ESBL family present or the specific bacterial species) (30).

Finally, accurate identification of ESBL-E can limit their dissemination. Reasons for the spread of ESBL-E genes include horizontal transfer of mobile genetic elements harboring ESBL genes, successful bacterial clones containing ESBL genes, ingestion of contaminated animal products, excessive antibiotic use, poor sanitation, and human migration patterns (23). Fewer investigations into risk factors for the dissemination of p-*ampC* have been conducted. Successful interventions to limit the relatively rapid spread of ESBL-E (e.g., decolonization strategies) cannot be executed in the absence of accurate identification.

Of note, the nomenclature for *ampC* genes, particularly c-*ampC* genes, is less standardized than that of ESBL genes, lending itself to difficulty in advancing our understanding of the prevalence, relatedness, and clinical impact of c-*ampC* genes across Enterobacterales species (12). As an example, the c-*ampC* gene of *K. aerogenes* has been named “*ampC*” by some investigators (31, 32) and *bla*_CMY_ by others (33, 34), despite generally sharing more than 95% nucleotide sequence identity. The clinical relevance of c-*ampC* genes is also more complicated than with p-*ampC* genes as their mere presence does not indicate whether these genes are expressed in an amount sufficient to hydrolyze broad-spectrum antibiotics (e.g., ceftriaxone). This is also the case for c-*ampC* genes present in *E. coli*; our data highlight that even in the presence of mutations in promoter or attenuator regions of the *E. coli ampC* gene, *ampC* expression may not be present.

Admittedly, while our study was designed to capture all Enterobacterales, certain species were represented in small numbers (e.g., *M. morganii* and *P. stuartii*), preventing us from drawing precise conclusions regarding their β-lactamase prevalence. As stated, our cohort only included the greater Maryland region, limiting its generalizability. Another important limitation is that the differentiation between certain “narrow spectrum” β-lactamase and ESBL enzymes is not clear, and absolute categorization as an ESBL or non-ESBL is not possible without additional enzymatic analysis. As an example, over 99% of *K. pneumoniae* isolates carry a chromosomal *bla*_SHV_ gene (14, 35). When the chromosomal *bla*_SHV_ gene is overexpressed, *K. pneumoniae* may exhibit an ESBL phenotype without the presence of a recognized ESBL gene (36). Similarly, there is controversy as to whether OXY-1 and OXY-2 are true ESBLs. Some data suggest that they may function more like narrow-spectrum β-lactamases unless they are overexpressed (36).

These limitations notwithstanding, there is value in understanding local ESBL and *ampC* prevalence to guide optimal antibiotic decision-making. Until there are significant advancements in comprehensive β-lactamase molecular diagnostics, antibiotic stewardship teams and clinical microbiology laboratories should partner to assist clinicians with recognizing the bacterial species most suggestive of ESBL and AmpC production to balance effective antibiotic prescribing with judicious antibiotic prescribing.

## SUPPLEMENTAL MATERIAL

Supplemental material aac.01258-24-s0001.xlsx Bacterial species, sequence types, plasmids identified, and antimicrobial resistance markers for 500 ceftriaxone-resistant Enterobacterales isolates.

ASM does not own the copyrights to Supplemental Material that may be linked to, or accessed through, an article. The authors have granted ASM a non-exclusive, world-wide license to publish the Supplemental Material files. Please contact the corresponding author directly for reuse.
